# Hitting HIV where it hides

**DOI:** 10.1186/1742-4690-5-15

**Published:** 2008-02-01

**Authors:** Andrew I Dayton

**Affiliations:** 1 Laboratory of Molecular Virology, Division of Emerging and Transfusion Transmitted Diseases, OBRR/CBER/FDA, HFM 315, 1401 Rockville Pike, Rockville, MD 20852-1448, USA

## Abstract

The recent finding that inhibitors of PI3/Akt can sensitize HIV infected macrophages to oxidative stress-induced cell death suggest a potential new therapeutic approach to targeting HIV reservoirs.

Although antiretroviral therapy has achieved laudable successes in combating HIV, particularly since the advent of protease inhibitors in the mid 1990s, fully successful treatment remains plagued by multiple, clinically latent viral reservoirs largely impervious to antiretroviral drugs. The microbiology of clinical latency is controversial and may involve elements of viral quiescence (expression of no – or a limited subset of – viral genes) as well as low level viral replication in protected cell types or anatomical compartments [1]. Viral persistence has been reported in brain cells (including perivascular macrophages, parenchymal microglial cells and astrocytes), NK cells, renal tubular cells, mononuclear cells from semen, follicular dendritic cells, cells of the monocyte/macrophage lineage (recently infected monocytes and tissue macrophages) and resting CD4+ T cells, with the latter two being the best known reservoirs [2-5].

Attempts at attacking the resting CD4+T cell HIV reservoir have generally involved induction (of presumably quiescent virus) with IL-2, IL-7, phorbol esters, or valproic acid [3,6,7]. Such induction approaches usually assume the activated, HIV producing cells will be killed directly by the induced virus or by the host immune system but some have attempted bolstering these effects by targeting immunotoxins to viral determinants [7]. The risk of a spreading infection by virus newly induced to replicate is generally mitigated in these scenarios by HAART.

Attacking the macrophage HIV reservoir has proven a thornier issue. From the virus's standpoint macrophages are an ideal reservoir cell because they are long lived, because HIV does not kill macrophages by direct lysis, as it does CD4+T cells, and because virus production by chronically infected macrophages tends to be relatively insensitive to a variety of antiretroviral agents [8-13]. Besides hosting a significant virus reservoir, chronically infected macrophages and/or their brain counterparts, microglia, may contribute to pathogenesis through chronic aberrant release of a variety of host and viral cytoactive factors and may be subject to chronic dysregulation through aberrant expression of surface receptors [14-20]. Thus, the recent report that PI3K/Akt inhibitors can drastically sensitize HIV infected macrophages to oxidative-stress-induced cell death [21] is welcome news as delineating a possible novel therapeutic approach.

HIV infection in vivo increases levels of superoxide anion and peroxynitrite, the latter of which can promote HIV replication in macrophages[22]. Recently Chugh et al. [23] reported that HIV infection activated the PI3K/Akt pathway exerting a cytoprotective effect against apoptotic challenge in a microglial cell line and in primary human macrophages. This described a pathway by which HIV could protect certain HIV infected cells against the oxidative stress they typically endure in vivo due to the high levels of nitric oxide (NO) they produce [24-27]. The finding that a variety of PI3K/Akt inhibitors, including wortmannin, Akt inhibitors IV & VIII (Calbiochem) and the clinically available Miltefosine could all promote cell death in cultures of primary human macrophages infected with HIV, but not in uninfected controls, makes therapeutically attacking the HIV macrophage/microglial reservoir a tantalizing possibility.

Recent work has contributed significantly to understanding the roles of numerous HIV regulatory proteins in cells of lineages other than the T lineage [22,28,29] and the work highlighted here is no exception. Mechanistic studies determined that the HIV Tat can mediate the activation of the PI3K/Akt pathway, dependent upon the Tat basic domain (a region that binds p53 [21,23]) and that the mediation is associated with a drop in the level of PTEN (phosphatase tensin homolog) protein expression. SIV Tat was also shown to mediate the cytoprotective effect (in a microglial cell line), suggesting an evolutionarily conserved role. The results are consistent with a model in which Tat competes with PTEN for p53 binding, causing p53 destabilization and a consequent reduction in PTEN mRNA and protein levels, relieving the PTEN inhibition of Akt activation (Figure 1).

**Figure 1:**
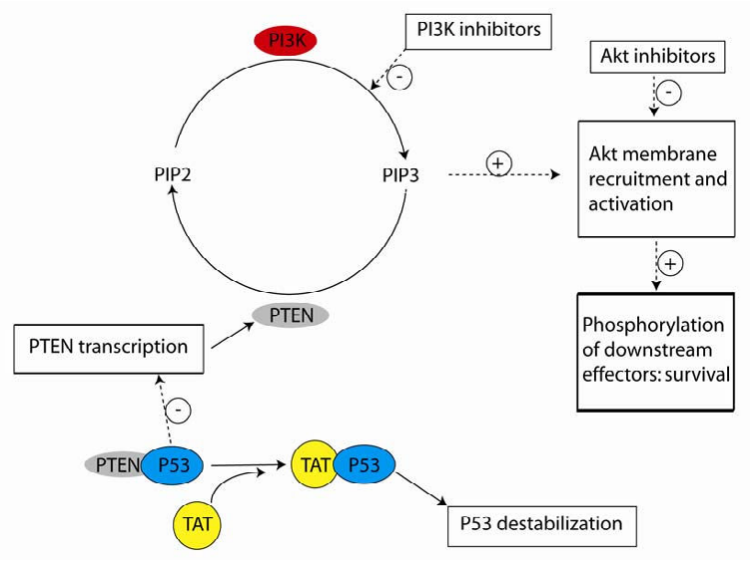
Proposed pathways [21] describing the effects of Tat and PI3K/Akt inhibitors on macrophage resistance to oxidative stress. Solid lines represent the flux of indicated molecular species. Dashed lines represent stimulatory (+) or inhibitory (-) regulation. Boxes enclose summaries of processes or effects.

Missing from the current in vitro findings is evidence that endogenous production of reactive oxygen species (ROS) in HIV infected macrophages or microglia is sufficient to render them more susceptible than uninfected control cells to oxidative stress-induced cell death [30,31]. Rather, exogenous NO must be provided in vitro (in the form of sodium nitroprusside) [21,23]. Thus, for the suggested approach to succeed clinically, either in vivo levels of ROS in critical local compartments must be sufficient to cause death when the Akt pathway is inhibited – the likelihood of which is undetermined – or such levels of ROS must be induced – which is problematic. Time will tell.
